# The Combined Dexamethasone/TSST Paradigm – A New Method for Psychoneuroendocrinology

**DOI:** 10.1371/journal.pone.0038994

**Published:** 2012-06-11

**Authors:** Julie Andrews, Catherine D’Aguiar, Jens C. Pruessner

**Affiliations:** 1 Douglas Mental Health University Institute, McGill University, Montreal, Quebec, Canada; 2 McGill Centre for Studies in Aging, Faculty of Medicine, McGill University, Montreal, Quebec, Canada; Wake Forest University, United States of America

## Abstract

The two main physiological systems involved in the regulation of the stress response are the hypothalamus-pituitary-adrenal (HPA) axis and the sympathetic nervous system (SNS). However, the interaction of these systems on the stress response remains poorly understood. To better understand the cross-regulatory effects of the different systems involved in stress regulation, we developed a new stress paradigm that keeps the activity of the HPA constant when exposing subjects to psychosocial stress. Thirty healthy male participants were recruited and randomly assigned to either a dexamethasone (DEX; n = 15) or placebo (PLC; n = 15) group. All subjects were instructed to take the Dexamethasone (2 mg) or Placebo pill the night before coming to the laboratory to undergo the Trier Social Stress Task (TSST). Salivary cortisol, salivary alpha amylase (sAA), heart rate, blood pressure and subjective stress were assessed throughout the protocol. As expected, the DEX group presented with suppressed cortisol levels. In comparison, their heart rate was elevated by approximately ten base points compared to the PLC group, with increases throughout the protocol and during the TSST. Neither sAA, nor systolic or diastolic blood pressures showed significant group differences. Subjective stress levels significantly increased from baseline, and were found to be higher before and after the TSST after DEX compared to placebo. These results demonstrate a significant interaction between the HPA and the SNS during acute stress. The SNS activity was found to be elevated in the presence of a suppressed HPA axis, with some further effects on subjective levels of stress. The method to suppress the HPA prior to inducing stress was found to completely reliable, without any adverse side effects. Therefore, we propose this paradigm as a new method to investigate the interaction of the two major stress systems in the regulation of the stress response.

## Introduction

For several decades now, psychoendocrine stress research has investigated the activation of the hypothalamus-pituitary-adrenal (HPA) axis in relation health and disease. It has been well established that psychological stress activates the HPA axis and its end product, cortisol, has become a reliable biological marker of the stress response [1]. Upon activation of the HPA axis, the hypothalamus releases corticotrophin-releasing hormone (CRH), which stimulates the release of adrenocorticotropic hormone (ACTH) from the anterior pituitary. This in turn triggers the adrenal cortex to release cortisol into the bloodstream [2].

The association between HPA axis regulation and various disease states has been studied extensively, and a dysregulation of the stress response (either a hyper- or hypoactivity) has been associated with a host of negative health outcomes, e.g., diabetes, hypertension, vascular disease [3], [4], [5].

Theories, such as the “allostatic load theory” describe possible mechanisms linking stress systems and disease. While a role of the stress systems in causing health ailments is rarely disputed, few studies have actually investigated the consequences of acute manipulation of the cortisol release in response to stress on physiological and psychological variables.

To investigate the acute stress response, one of the most frequently used laboratory stressors is the Trier Social Stress Task [1], [6]; a combination of a free speech and mental arithmetic in front of an audience. The typical cortisol stress response to the TSST has been well described, with a cortisol peak occurring 20–30 minutes after stressor onset and hormone levels returning to baseline within sixty minutes after stress onset [1], [7]. This delivery of the cortisol stress hormone is believed to aid the organism in dealing with the increased energy demands that the situation requires [8].

Other systems change their activity in response to a stressor as well. Aside from the HPA axis, the sympathetic nervous system (SNS) is prominently involved in the stress response [9]. The result of an activation of the SNS system are changes in heart rate, blood pressure, galvanic skin response, salivary alpha-amylase (sAA) [10], among others. The two systems are not equally activated in response to stress, and recent studies demonstrate that the HPA axis response is stronger to social-evaluative types of stress, while the SNS is stronger in response to stressors causing anxiety and fear [8]. These measures are thus frequently included in stress studies. It can be expected that there is a cross-talk between the two stress systems, i.e. that they interact in relation to the perception and processing of psychosocial stress. However, no systematic studies have looked at this interaction - specifically what happens to one system if the other one failed to respond has to the best of our knowledge not been previously investigated.

The aim of the present study was thus to investigate the interaction between the HPA and SNS systems by blocking the acute HPA axis response and then exposing the subject to an acute stressful situation. The research question led to the development of a new stress paradigm: a combination of the dexamethasone suppression test (DST) and the TSST, termed “The combined Dexamethasone/TSST paradigm.”

The DST is commonly used to test negative feedback inhibition of the HPA axis. As a potent synthetic glucocorticoid (GC), dexamethasone (DEX) primarily binds to GC receptors in the periphery, and the pituitary [11], [12], [13], [14], [15], resulting in an almost complete suppression of pituitary release of ACTH for several hours, lasting into the morning following DEX administration the evening before [12]. The lack of ACTH then leads further to almost complete absence of cortisol since the adrenal cortex is not stimulated. It is important to differentiate the effects of a low to moderate administration of DEX between the brain and the periphery. DEX does not cross the blood-brain barrier and thus will not reach receptors above the level of the pituitary in the central nervous system [12], [16], thus depriving the CNS from any stimulation with glucocorticoids, and causing a hypocorticoid state. In contrast, the amounts typically used in the DST (1–2 mg) lead to a flooding of receptors in the periphery, causing a hypercorticoid state in the body. In the combined Dexamethasone/TSST paradigm, subjects are exposed to the TSST after DEX has been given the night before, and thus this paradigm allows to test the response of the individual to an acute and strong stressor at the psychological level (effects on the perception of the stressor) and the physiological level (effects on the activity of the SNS), in the absence of an HPA axis stress response. Given the role of the HPA system in coping with stress, and the interaction between the different stress systems in the human organism, we hypothesized that the blockade of a cortisol response to acute stress will result in an increased perception of psychological stress, and an increased activity of the SNS.

## Methods

### Ethics Statement

McGill University’s Faculty of Medicine Institutional Review Board approved this protocol. All subjects provided written informed consent prior to entering the study.

### Subjects

Since this was to the best of our knowledge the first study of its kind to investigate the interaction between the stress systems we decided to focus our study on men, to avoid a confounding effect of cycling menstrual hormones on the HPA and SNS systems. Thirty healthy men between the ages of 18 and 35 (*M* = 24.0±5.17) with normal body mass index (*M* = 22.22±5.17) were recruited through classified ads. All participants were screened over the phone for their medical history. Subjects with medical or psychiatric conditions (e.g. diabetes, depression, etc.) or taking medications, known to affect endocrine function, such as glucocorticoids, were excluded. Smokers were also excluded as smoking affects the HPA axis [1].

### General Procedure

Participants were randomly assigned to either of two conditions: (1) placebo (n = 15, mean age = 23.80±5.20) and (2) dexamethasone (DEX) (n = 15, mean age = 22.20±5.32). Two appointments were made with each subject. The first appointment served to explain the study procedures, complete the consent form, and to supply the participant with the study materials: either placebo, or 2 mg DEX in the form of a tablet to be taken orally. The experimenter then informed the subject to take the pill at bedtime the night before the testing session at our laboratory. The second appointment then took place at 10h00 or 10h30 a.m., the morning after DEX administration. Participants arrived at the laboratory at least an hour before the stress session and completed a battery of questionnaires assessing numerous personality traits known to affect stress reactivity including parental care [17], self-esteem [18], [19], and depressive symptomatology [20], [21]. After an additional 40-minute rest period, participants were exposed to the TSST [1]. Participants then rested for another 40 minutes, and were then debriefed about the nature of the study.

In this study we opted for a *between subject* design because of the known habituation effects of repeated stress testing exposure. Previous studies have shown that participants may habituate to multiple exposures of the TSST [22], [23], [24], which would then confound our effects when comparing the DEX and PLC groups.

### Psychological Stress Induction

In this study, a slightly modified version of the TSST was used. Participants were brought to a waiting room adjacent to the testing room and instructions were read out. Subjects were instructed that they would be doing a mock job interview and had 5 minutes to convince a panel of behavioral experts that they were the best candidates for the job. The experimenter introduced the panel, informed the participants that they would be evaluating their verbal and paraverbal skills and that their speech would be recorded via camera for a later more in-depth analysis. Moreover, after the 5-minutes interview, the committee would let them know of a second task. The subjects were then given ten minutes to prepare for their presentation (anticipation phase). In the current study, an extra 5-minute period was inserted between the anticipation phase and the presentation to allow the participants to fill out additional questionnaires, including the Primary Appraisal and Secondary Appraisal questionnaire [25], a modified version of the Fear of Negative Evaluation Scale [26] and COPE inventory [27].

### Physiological and Endocrinological Measures

**Figure 1:**
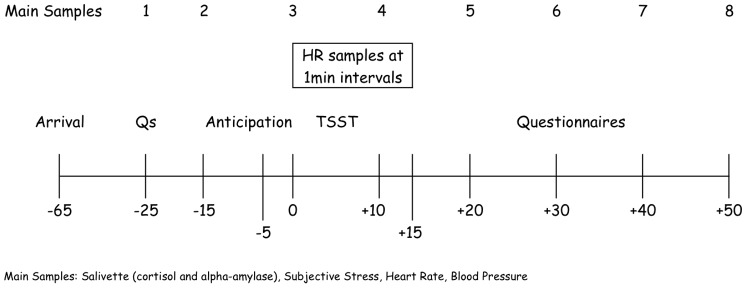
Timeline of the testing procedure.

Saliva samples were collected using salivettes (Sarstedt, Quebec City, Quebec, Canada) to assess cortisol and alpha-amylase. Cortisol was analyzed with a time-resolved fluorescence immunoassay with proven reliability and validity [28]. Alpha-amylase was analyzed via the enzyme kinetic method outlined elsewhere [29]. Blood pressure and heart rate were measured by an ambulatory (A&D Company, Tokyo, Japan) before the TSST, and finger pulse oximeter (Roxon, Montreal, Canada) during the TSST. Prior to statistical analysis, readings from the finger pulse oximeter and the sphygmomanometer were synchronized, using the latter as the reference value. Finally, subjective stress was evaluated using a ten-point visual analogue scales asking “How stressed do you feel right now?” ranging from “not at all” to “extremely.” All measures were assessed in 10 to 15 minute intervals throughout the protocol. Additionally, heart rate was assessed in 1-minute intervals during and 5-minutes post TSST. Figure 1 depicts the timing of each sampling.

### Statistical Analysis

Serial one-way (Group) analysis of variance (ANOVAs) with age, body mass index (BMI) and several personality factors as dependent variables were conducted to ensure the groups did not differ on these variables. Analyses for cortisol, alpha-amylase, heart rate, blood pressure and subjective stress were carried out.

To investigate the interaction and main effects of the experimental conditions on the stress response on the cortisol levels over time a two-factor (Time x Group) between-design repeated ANOVA with the eight cortisol measures as dependent variable. This analysis was repeated for alpha-amylase, heart rate, subjective stress ratings, systolic and diastolic blood pressure, with the eight respective values as the (repeated measures) dependent variable. We also performed this analysis with the fifteen one-minute heart rate measures during and after the TSST.

In cases where assumptions of sphericity were violated, Huynh-Feldt corrections were performed. This correction adjusts the degrees of freedom in the ANOVA test and consequently the significance (p) value. Additionally, post-hoc analyses using Tukey’s Honestly Significant Difference (HSD) test were conducted on significant results. This test was performed to understand the direction of the significant differences and pinpoint exactly between which timepoints the differences were observed.” All statistical analyses were completed with the SPSS software package16.0.1 for Apple OS X software version 10.5.8.

## Results

The serial one-way (Group) ANOVAs revealed no significant differences between the groups for age, BMI and any of the personality variables (all *Fs*<3.03, *ps*>0.05). These results suggest that both groups were comparable on these factors and that they were unlikely to contribute to endocrinological, cardiovascular or cognitive differences observed between the groups.

### Effects of the Experimental Conditions on Salivary Cortisol

The two-factor (Time x Group) between-design repeated ANOVA showed a significant interaction effect of Time x Group, F_(2.07, 57.96)_ = 8.28, p = 0.001, and a main effect of Time, F_(2.07, 57.96)_ = 8.82, p<0.0001, and, as expected, a highly significant effect of Group, F_(1, 28)_ = 91.08, p<0.0001.

To further explore the interaction and main effects, we performed a Tukey HSD. This analysis revealed significant differences at all timepoints between the DEX and PLC groups, with all cortisol levels in the DEX group lower than in the PLC group, all p<0.001.

**Figure 2:**
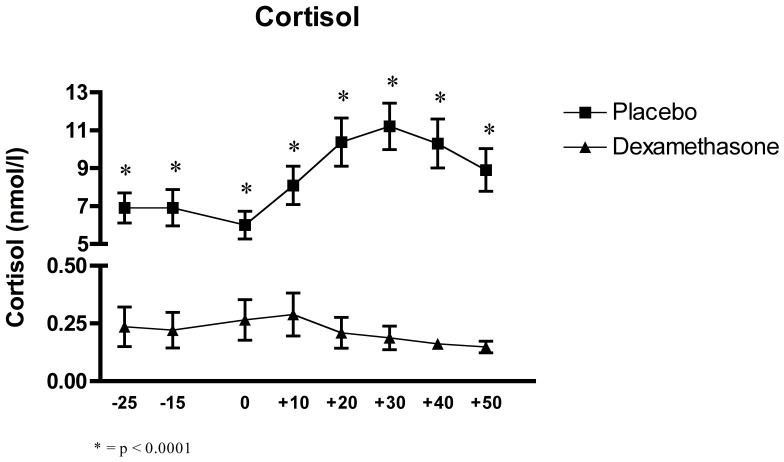
Effects of the TSST on the cortisol release in relation of the two experimental conditions: dexamethasone (n = 15) and placebo (n = 15).

Furthermore, in the PLC group, the *Post-Hoc* analysis revealed significant differences between samples 1 through 3 (pre-TSST) and 5 through 7 (post-TSST and recovery), indicating that the baseline measures were lower than the other values, all p<0.001. Sample 4 (post-TSST) was also found to be significantly lower than samples 5 and 6 (post-TSST), all p<0.05. In contrast, no significant differences were observed between any of the samples in the DEX group (see Figure 2). Throughout the test, the DEX group had cortisol levels which were at or below the sensitivity threshold of the assay, indicating an almost complete suppression of the HPA axis.

### Effects of the Experimental Conditions on Subjective Stress

The analysis of the subjective stress with a two-factor (Time x Group) between-design repeated ANOVA showed a significant interaction effect of Time x Group before the Huynh-Feldt correction, F_(7, 196)_ = 2.357, p = 0.025 and a trend post correction, p = 0.077. A significant main effect of Time was also observed, F_(3.018, 84.501)_ = 45.227, p<0.0001, but no main effect of Group, F_(1, 28)_ = 0.723, p>0.05.

**Figure 3:**
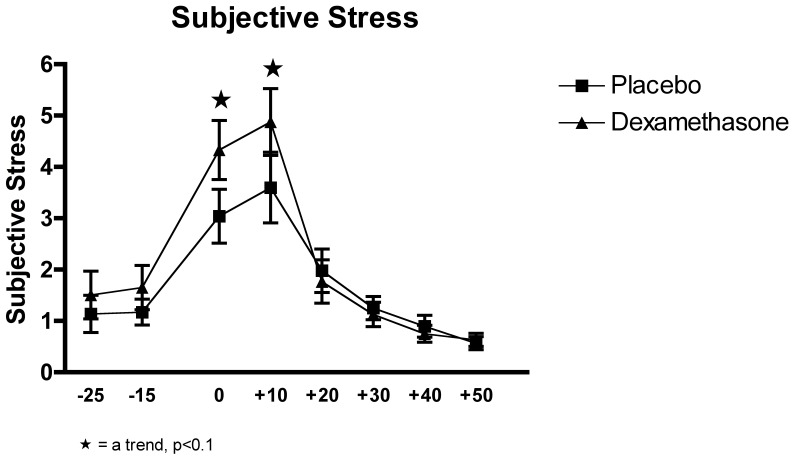
Effects of the TSST on the subjective stress ratings in relation of the two experimental conditions: dexamethasone (n = 15) and placebo (n = 15).

A trend was observed at timepoint 3 and 4 between the groups, p<0.1, where the DEX group showed higher subjective ratings. Taken together, this set of findings suggested that there was at least a trend for higher stress perception in the DEX group, particularly during the exposure to the TSST. (see Figure 3).

### Effects of the Experimental Conditions on Salivary Alpha-Amylase

The analysis of the effect of the condition on the salivary alpha-amylase conducted by the two-factor (Time x Group) between-design repeated ANOVA solely revealed a significant main effect of Time, F_(4.41, 123.34)_ = 12.52, p<0.001, but neither a significant interaction effect of Time x Group, nor a main effect of Group, all Fs<1, p>0.5, thus suggesting that the complete suppression of HPA axis activity did not have a significant effect on sAA levels.

**Figure 4:**
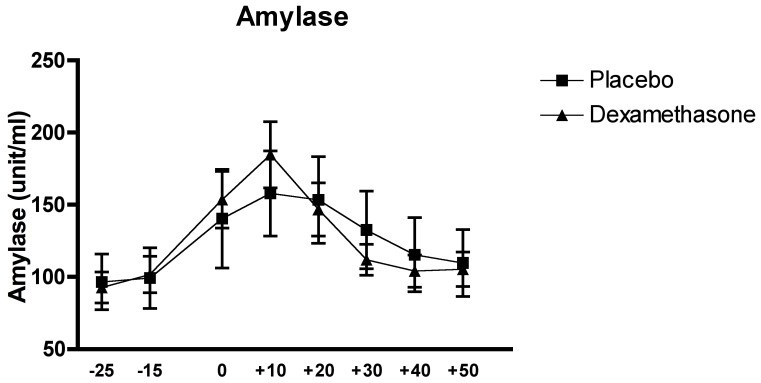
Effects of the TSST on the salivary alpha-amylase in relation of the two experimental conditions: dexamethasone (n = 15) and placebo (n = 15).

The *Post-Hoc* analysis of the Time effect revealed significant differences, with samples being higher during the acute stress exposure (all p<.001). These effects illustrate the increase in sAA levels in response to the TSST. (see Figure 4).

### Effects of the Experimental Conditions on Heart Rate

**Figure 5:**
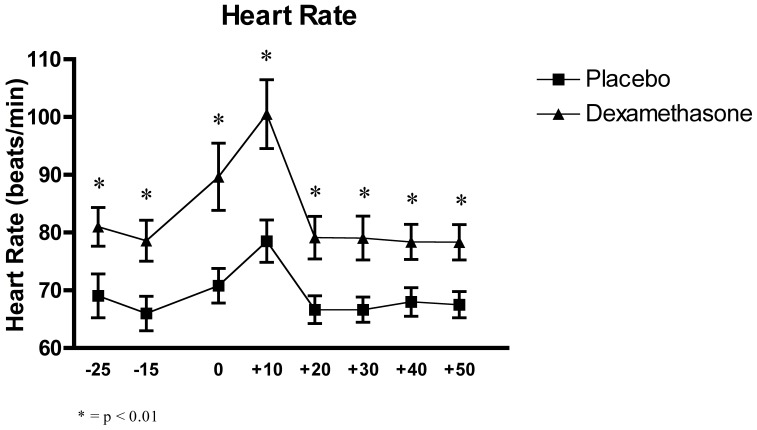
Effects of the TSST on the heart rate in relation of the two experimental conditions: dexamethasone (n = 15) and placebo (n = 15).

The two-factor (Time x Group) between-design repeated ANOVA with the eight main heart rate measures showed a significant interaction effect of Time x Group, F_(4.28, 119.78)_ = 3.13, p = 0.015, and a main effect of Time, F_(4.28, 119.78)_ = 25.88, p<0.0001, and Group, F_(1, 28)_ = 9.23, p = 0.005. Post-hoc tests indicated that while both groups showed an increase of heart rate during the acute stress induction (all p<.001), the DEX group was significantly higher than the PLC group, in all samples (all p<0.0012), by about ten beats per minute (see Figure 5).

Furthermore, the two-factor (Time x Group) between-design repeated ANOVA with the fifteen 1-minute interval heart rate measures confirmed a significant interaction effect of Time x Group, F_(6.94, 194.44)_ = 2.03, p = 0.053, and a main effect of Time, F_(6.94, 194.44)_ = 12.85, p = 0.0001, and Group, F_(1, 28)_ = 11.08, p = 0.003.

Here, post-hoc analysis revealed that there was a further increase in heart rate in the middle of the stress session as compared to the recovery period, and that samples from the DEX group were found to be significantly higher than the PLC group throughout (all p<.05).

**Figure 6:**
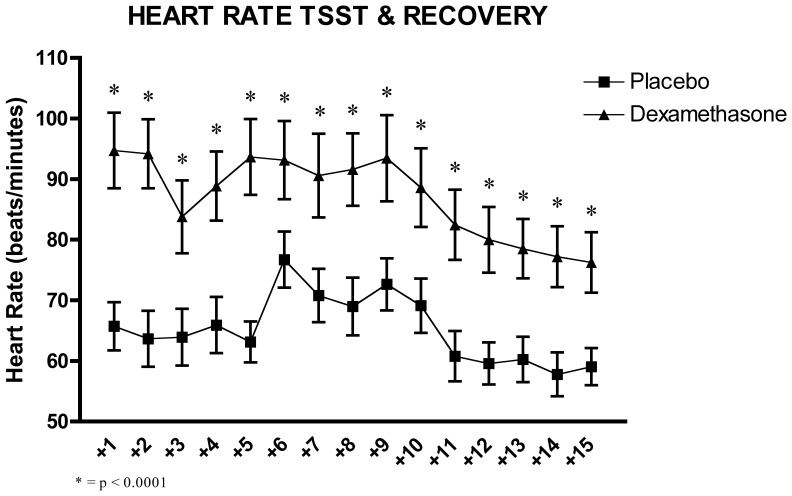
Effects of the TSST on the heart rate in 1-minute intervals during and 5 minutes post-TSST in relation of the two experimental conditions: dexamethasone (n = 15) and placebo (n = 15).

Taken together, these effects illustrate that one main effect of suppressing DEX was a higher heart rate throughout the experiment, with higher levels at the beginning and throughout the experiment, but in addition higher levels also at the peak stress period during the TSST (see Figure 6).

### Effects of the Experimental Conditions on Blood Pressure

The analysis of the systolic and diastolic blood pressure with the two-factor (Time x Group) between-design repeated ANOVA, did not reveal a significant interaction effect of Time x Group or main effect of Group for both variables, all Fs<1.11, all ps>0.3. However the ANOVAs did show a significant Time effect for both variables, F_(4.99, 139.69)_ = 21.32, p<0.0001, for the systolic blood pressure and F_(6.70, 187.71)_ = 23.95, p<0.0001 for the diastolic blood pressure.

**Figure 7:**
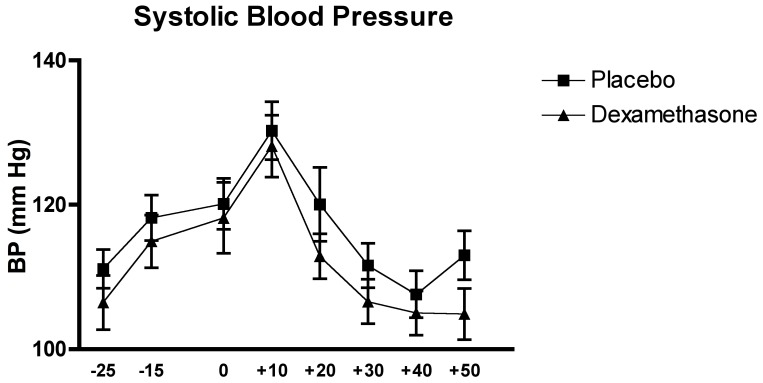
Effects of the TSST on the systolic blood pressure in relation of the two experimental conditions: dexamethasone (n = 15) and placebo (n = 15).

**Figure 8:**
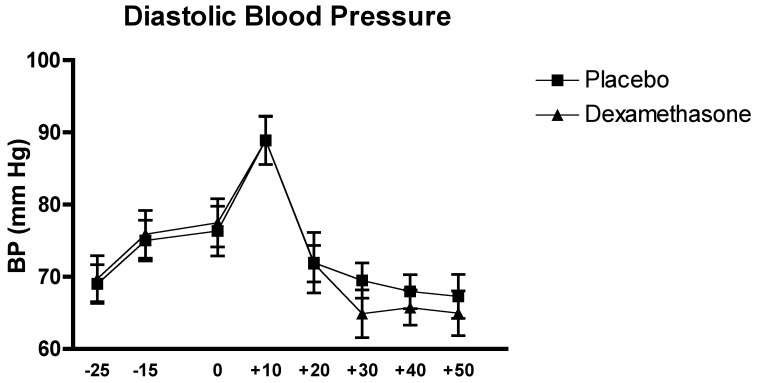
Effects of the TSST on the diastolic blood pressure in relation of the two experimental conditions: dexamethasone (n = 15) and placebo (n = 15).

The *Post Hoc* analysis of the Time effect of the systolic blood pressure revealed that systolic blood pressure levels were higher during the acute stress exposure (all p<0.02). The post hoc analysis for the diastolic blood pressure Time effect showed a peak directly after the TSST exposure, when compared to baseline and recovery (all p<0.005 (see Figure 7 and 8).

## Discussion

The goal of the present study was investigate the effects of suppressing the HPA axis system in the presence of an acute stressor on blood pressure, heart rate, alpha-amylase, and the subjective experience of stress. In order to do so, we applied an HPA axis suppressant prior to administering acute psychosocial stress. The combined Dexamethasone/TSST paradigm was successful in investigating the effects of HPA axis suppression on salivary alpha-amylase, subjective stress, heart rate and blood pressure. To the best of our knowledge no previous studies have investigated the effect of suppressing the HPA axis response during an acute psychological stress on levels of sAA, heart rate, blood pressure and subjective stress.

While the DEX group had cortisol levels at or below the detection threshold of the assay, both groups showed an increase in subjective stress ratings with a rise in response to the anticipation period, and the actual TSST, this result being in line with Hellhammer and Schubert [30], who also recorded subjective stress during the TSST. The peak level of subjective stress was close to peak levels in sAA, heart rate and blood pressure responses, suggesting that the subjective stress rating is more closely associated with the activity of the SNS system rather than the HPA. This is best demonstrated in the DEX group, which, in the absence of any HPA response showed a trend for higher subjective stress responses, in line with a higher heart rate. One possible explanation for this is the fact that the changes of the SNS are tangible, for example an increase in heart rate, while there are no known perceptible physiological changes related to a change in cortisol secretion.

The analysis of sAA showed a significant effect of Time with a typical response, peaking 10-minutes after onset of the stressor [31], [32], similar to the systolic and diastolic blood pressure analysis. In the absence of any group or group by time effect, it is relatively safe to conclude that the presence or absence of cortisol did not affect either sAA or blood pressure. When interpreting a non-significant result it is important to consider the power to find an effect in the study sample if it was indeed existing in the population. Thus, a post-hoc power analysis was conducted and demonstrated that, in this study, when assuming a medium effect size (*f* ^2^ = 0.25) we had a power of at least (1-β) = 0.96. This result dramatically decreased to (1-β) = 0.29 when assuming a small effect size (*f* ^2^ = 0.1) [33].

The most significant effect from HPA axis suppression, and thus from the entire study, was found in the change of heart beat throughout the experiment. The results showed significantly higher beats per minute in the DEX group compared to the PLC group, from the beginning of our heart rate recording, throughout the entire experiment: before, during and after the TSST. The effect was rather stronger, with a ten base point difference between the experimental groups.

There are a number of possible explanations for this effect. For one, it may be due to the central hypocortisol state created by the DST. The lack of negative feedback in the paraventricular nucleus of the hypothalamus will likely result in a CRH surge. It has been shown that there is a CRH-induced sympathetic activation, resulting in increased heart rate, blood pressure and glucose [34], [35]. Morphologically, CRH-secreting neurons from the hypothalamus project to the hindbrain and vice-versa. Also, the HPA and SNS seem to activate one another in a feed forward mechanism, via the CRH and locus coeruleus/norepinephrine (LC/NE) connection. Moreover, it has been shown that NE potentiates the release of CRH and application of CRH to the LC neurons increases its firing rate, further confirming the physiological link between the two systems (for review see [34]). Therefore, the hypocortisolemic state of the brain may be the cause for the elevated heart rate via a CRH surge, and the PVN and LC connection. Unfortunately, in the absence of any CRH measures this explanation must remain speculatory. Additionally, heart rate is mediated through the balance of the SNS and parasympathetic nervous system. Therefore, a possible effect of Dexamethasone via the parasympathetic nervous system could also be hypothesized.”

A second explanation for the elevated heart rate could be the direct effect of dexamethasone on peripheral tissue involved in cardiovascular regulation. Glucocorticoid (GC) receptors are found throughout the body including skin, lungs and heart [36]. It has been long recognized that steroids have an effect on cardiovascular function, and DEX has been shown to directly affect the beating of myocardial cells [36]. GCs also affect vasoconstriction and, interestingly, DEX also potentiates the pressor response to NE [37]. It must be noted that these effects do not directly alter heart rate, but it shows an involvement of GCs in cardiovascular regulation. As a third possible explanation, the higher heart rate found in the dexamethasone group may be due to a combination of both the hypo- and hyper-cortisol state in the CNS and the periphery working together, as the central hypocortisol state results in increased NE release and the peripheral hypercortisol state then could potentiate this effect.

Maheu et. al. (2005) also manipulated the HPA axis using metyrapone and observed an elevated heart rate pre and post-TSST. However, one must note the differential effects of metyrapone and DEX. As previsouly stated, DEX creates 2 states: a central hypocortisol state and a peripheral hypercortisol state, while metyrapone results in an overall hypocortisol state as it prevents the production of cortisol altogether. As these manipulations both result in an elevated heart rate, the most likely explanation for this would be their common effect on the central hypocortisol state. Even though, any hypotheses on the link between dexamethasone and cardiovascular effects have to be considered speculatory, as the exact mechanisms between the two are still unknown.”

Yet, we must note that indices of higher SNS activity found in the experimental group compared to the control group were restricted to heart rate, and not observed for our other SNS measures. The lack of group difference observed for the sAA measures may be explained by recent data suggesting that it is not a specific marker of SNS activity, but rather an overall measure of changes in the autonomic nervous system [38].

Overall, these findings may be explained by the fact that all of the employed measures for SNS were indirect. Therefore, effects on these indirect SNS markers may have been less consistent, resulting in smaller effect sizes, and thus harder to detect compared to measuring the SNS more directly (for example, through epinephrine and norepinephrine levels, which are however notoriously difficult to obtain by themselves). Previous literature has shown that blood pressure and heart rate changes do not occur always in parallel and that even when pharmacologically manipulating the SNS directly, changes are not always observed in all of these indirect measures. For example, after propranolol administration, a beta-blocker, Maheu et. al. [39] observed no significant differences in heart rate and diastolic blood pressure after the TSST, when compared to the placebo group. However, they observed significant lower systolic blood pressure. These findings demonstrate that even when directly affecting the SNS not all indirect biomarkers will show change. Similarly, sAA may not be a specific marker of SNS activity, but rather an overall measure of change in the autonomic nervous system, further confounded by flow rate, as already mentioned. Moreover, it has been shown that a chronically elevated heart rate may cause vasoconstriction, arterial stiffening and insulin resistance, all resulting in hypertension [40]. However in this study, the elevated heart rate state may have been too acute and could explain why the effect is not reflected in the blood pressure measures. The low and single dose of DEX may also explain why we do not observe a significant blood pressure difference between the groups, as Brotman et. al. [41] observed an elevated blood pressure after a 5 day, 3 mg twice a day regiment of dexamethasone. Nonetheless, despite the lack of group difference, both variables did show a typical pattern of stress reactivity.

Taken together, the combination of Dexamethasone with the TSST paradigm allowed us to investigate the interaction between the various stress systems by suppressing the HPA. This task may be used to further examine and disentangle the contribution of each of these systems in disorders involving a dysregulation of either of these systems, such as chronic stress, or the metabolic syndrome. In conditions where one of the available physiological stress systems is chronically changed, it would be very informative to investigate what effect such a change has on the complimentary stress response systems. We suggest that the DEX/TSST paradigm will allow you to that. In addition, other known effects of stress like memory, cognition, attention and decision making, could also benefit from the application of this paradigm to differentiate the contribution of the physiological versus the psychological systems in these previously observed effects. Future studies could also expand this line of research by performing the reverse test, i.e. by suppressing the SNS to investigate the effect on the HPA axis by using an appropriate SNS inhibitor like propranolol; this study is currently being conducted in our laboratory.

In conclusion, this study demonstrated that the SNS clearly responded differentially to a standardized stress paradigm in the presence or absence of an HPA axis response. When the HPA was suppressed, a significantly higher heart rate response to the TSST occurred, indicating an inverse relationship between the two systems, where SNS activity may be elevated in the presence of a blunted HPA axis response. An overactive SNS has previously been linked to hypertension, atherosclerosis, increased cardiovascular risk and events [40], [42]. Knowing that several psychopathologies, such as depression and burnout, are linked with the dysregulation of the HPA, this finding may further have critical health implications. It is unclear whether the interaction between the HPA and the SNS is continuous, i.e. whether a more subtle blunting of the HPA would similarly result in a more slightly elevated heart rate response. However, given the high prevalence of cardiovascular diseases in the developed world, this clearly deserves further investigation.
